# Norrin protects optic nerve axons from degeneration in a mouse model of glaucoma

**DOI:** 10.1038/s41598-017-14423-8

**Published:** 2017-10-27

**Authors:** Stephanie A. Leopold, Ludwig F. Zeilbeck, Gregor Weber, Roswitha Seitz, Michael R. Bösl, Herbert Jägle, Rudolf Fuchshofer, Ernst R. Tamm, Andreas Ohlmann

**Affiliations:** 1 Institute of Human Anatomy and Embryology, University of Regensburg, Regensburg, Germany; 2 Experimental Biomedicine, University of Würzburg, Würzburg, Germany; 3 University Eye Hospital, University of Regensburg, Regensburg, Germany; 4 Department of Ophthalmology, Ludwig-Maximilians-University Munich, Munich, Germany

## Abstract

Norrin is a secreted signaling molecule activating the Wnt/β-catenin pathway. Since Norrin protects retinal neurons from experimental acute injury, we were interested to learn if Norrin attenuates chronic damage of retinal ganglion cells (RGC) and their axons in a mouse model of glaucoma. Transgenic mice overexpressing Norrin in the retina (Pax6-Norrin) were generated and crossed with DBA/2J mice with hereditary glaucoma and optic nerve axonal degeneration. One-year old DBA/2J/Pax6-Norrin animals had significantly more surviving optic nerve axons than their DBA/2J littermates. The protective effect correlated with an increase in insulin-like growth factor (IGF)-1 mRNA and an enhanced Akt phosphorylation in DBA/2J/Pax6-Norrin mice. Both mouse strains developed an increase in intraocular pressure during the second half of the first year and marked degenerative changes in chamber angle, ciliary body and iris structure. The degenerations were slightly attenuated in the chamber angle of DBA/2J/Pax6-Norrin mice, which showed a β-catenin increase in the trabecular meshwork. We conclude that high levels of Norrin and the subsequent constitutive activation of Wnt/β-catenin signaling in RGC protect from glaucomatous axonal damage via IGF-1 causing increased activity of PI3K-Akt signaling. Our results identify components of a protective signaling network preventing degeneration of optic nerve axons in glaucoma.

## Introduction

Norrin is a secreted protein with some structural similarities to secreted ligands of the TGF-β family^1–3^. In the postnatal retina, Norrin is primarily expressed in Müller glia^4,5^ and functions as a high affinity frizzled-4 ligand^6^, which activates canonical Wnt/β-catenin signaling upon binding the co-receptor Lrp5^5,6^. The transmembrane protein TSPAN12 promotes the formation of the Norrin/Fz4/Lrp5 signaling complex^7^. During development, intact Norrin/Fz4/Lrp5 signaling in vascular endothelial cells is an essential requirement for the formation of the retinal capillary networks in the outer and inner plexiform layers^5,6,8–10^. Moreover, Norrin protects from vascular injury during oxygen-induced retinopathy^11^, an effect that is mediated via the induction of insulin-like growth factor (IGF)-1^12^.

In the retina, activation of the canonical Wnt/β-catenin pathway has been implicated in protection and regeneration of retinal pigment epithelium cells and retinal ganglion cells (RGC) including their axons after acute injury^13,14^. Moreover, prolonged Wnt/β-catenin signaling leads to enhanced proliferation of Müller cells expressing markers for retinal progenitor cells^15^. The retinal expression of Norrin in the mammalian retina continues throughout adult life^16,17^, and there is evidence for a role of Norrin in maintenance and protection of retinal neurons. Photoreceptors of transgenic mice with overexpression of Norrin in cells of the retinal pigment epithelium are protected against light-induced damage^18^. In this model of acute photoreceptor injury and subsequent apoptosis^19^, the protective role of Norrin is mediated via canonical Wnt/β-catenin and endothelin-2 (EDN2) signaling, and involves the neuroprotective effects of brain-derived neurotrophic factor and PI3K-Akt^18^. Moreover, recombinant Norrin protects RGC from excitotoxic damage induced by the intravitreal injection of NMDA^20^, a model of acute RGC cell injury^21^. Again, canonical Wnt/β-catenin and EDN2 signaling are involved, which increase in Müller cells the secretion of the neuroprotective molecules leukemia inhibitory factor (LIF) and fibroblast growth factor-2 (FGF-2)^20^.

In this study we were interested to learn if Norrin is capable of protecting RGC in glaucoma, a neurodegenerative disease and frequent cause of blindness worldwide^22,23^. To this end, we used the DBA/2J mouse model of hereditary glaucoma^24–26^ and generated DBA/2J mice with transgenic overexpression of Norrin in RGC. The critical risk factor in glaucoma is an intraocular pressure that is too high for the health of RGC leading to their damage and apoptosis^22,23^. There is evidence from studies of monkeys with experimental glaucoma^27^ and the DBA/2J mouse strain with hereditary glaucoma^28,29^ indicating that the primary site of glaucomatous RGC insult is at the optic nerve head where RGC axons leave the eye to project to the brain. Here we provide evidence that the overexpression of Norrin leads to the attenuation of chronic degeneration of optic nerve axons in DBA/2J hereditary mouse glaucoma. The protective effects correlate with constitutive activation of canonical Wnt/β-catenin signaling in RGC, an increase in the retinal amounts of IGF-1 and the enhanced activity of PI3K-Akt signaling. Our findings identify components of a protective signaling network with the potential to attenuate or prevent RGC axonal degeneration in glaucoma.

## Results

### Overexpression of Norrin in the sensory retina

**Figure 1:**
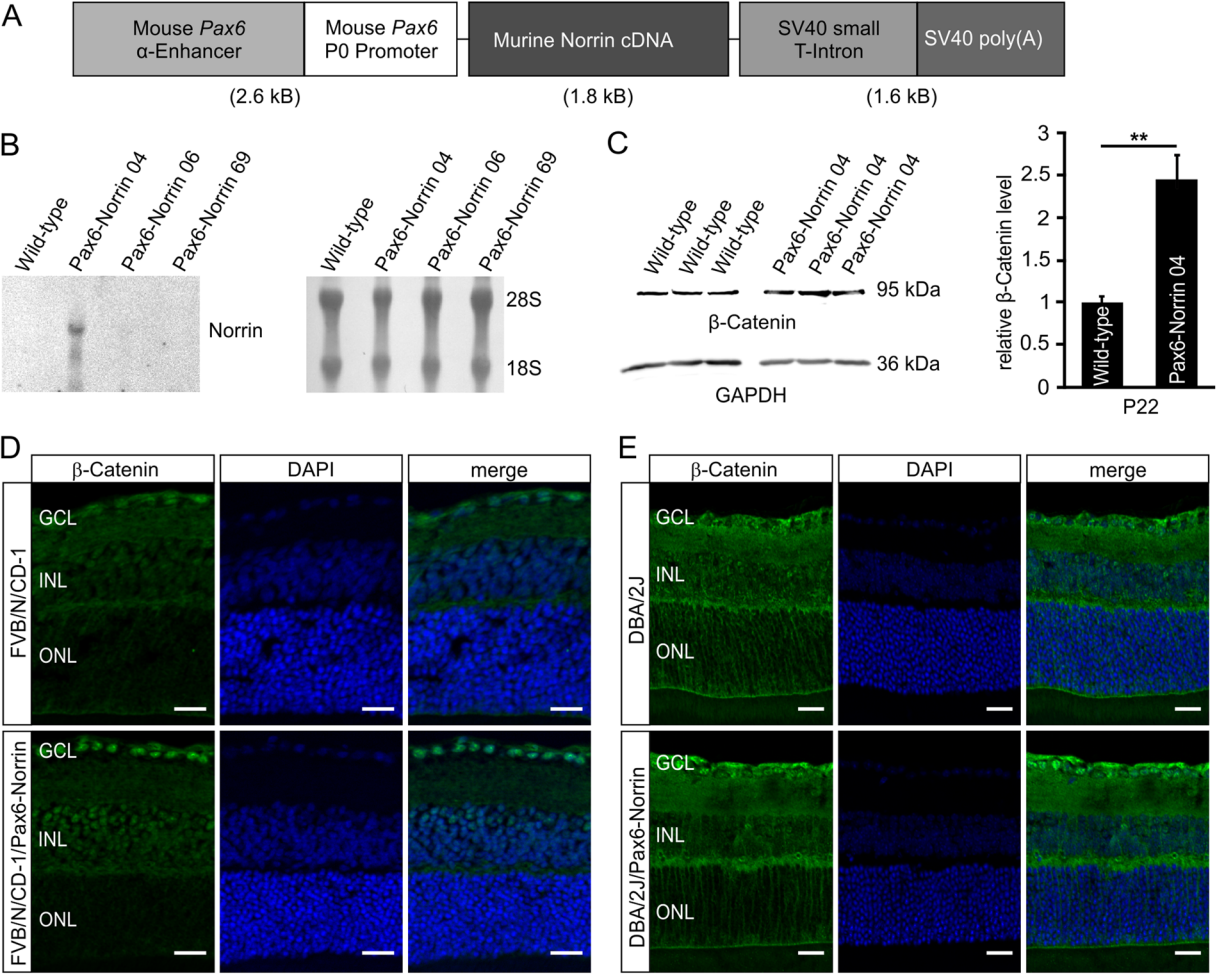
Generation of transgenic Pax6-Norrin mice. (**A**) Schematic drawing of the transgene. (**B**) Northern blot analysis of Norrin mRNA in RNA of retinae from three transgenic lines (Pax6-Norrin-04, -06 and -69) and wild-type littermates (WT) at P2. Integrity of loaded RNA was controlled by methylene blue staining. (**C**) Western blot analysis for β-catenin in retinae from transgenic line Pax6-Norrin-04 and wild-type littermates (WT) at P22. β-catenin levels were analyzed by densitometry, normalized to GAPDH and plotted as x-fold to levels of wild-type controls (mean ± SEM; n = 9; **p < 0.01). (**D,E**) Immunostaining for β-catenin (green) in P22 retinae of wild-type and transgenic Pax6-Norrin-04 mice in the FVB/N/CD-1 background (**D**) and crossed with DBA/2J mice (**E**). Nuclei are labeled with DAPI (blue). GCL. ganglion cell layer; INL. inner nuclear layer; ONL. outer nuclear layer; Scale bars: 20 μm.

To generate an animal model with constitutive overexpression of Norrin in the sensory retina, transgenic mice with a 1.8 kb murine Norrin cDNA under control of a murine *Pax6* fusion promoter (Pax6-Norrin) were generated (Fig. 1A). In the eye, the promoter fragment that consists of the 1.8 kb α-enhancer element upstream of the *Pax6* minimal promoter P0 promotes specific transgenic expression in cells derived from the inner layer of the optic cup^30–33^. After microinjection, three transgenic founder lines (Pax6-Norrin-04, -06 and -69) were generated. By northern blot analysis at postnatal (P) day 2 before endogenous Norrin expression starts, no signal for Norrin mRNA was detected in retinae of wild-type controls and lines Pax6-Norrin-06 and -69, indicating no or very weak transgenic Norrin expression (Fig. 1B). However, in the transgenic mouse line Pax6-Norrin-04, a specific and robust hybridization signal was detected strongly suggesting transgenic overexpression of Norrin in the sensory retina (Fig. 1B). The line (hereinafter referred to as Pax6-Norrin) was used for further studies.

We analyzed, if the transgenic expression of Norrin mRNA leads to an enhancement of canonical Wnt/β-catenin signaling in the inner parts of the retina. By western blot analysis in retinal proteins from three-week-old Pax6-Norrin mice, a significant 2.4-fold increase (p < 0.01) was observed compared to wild-type littermates (Fig. 1C). By immunohistochemistry, we detected on retinal meridional sections of wild-type mice a faint signal for β-catenin in nuclei of the retinal ganglion cell (RGC) layer. In contrast, in retinae from transgenic Pax6-Norrin littermates a pronounced nuclear staining for β-catenin in the RGC layer and in the inner nuclear layer was observed (Fig. 1D). Overall, our data strongly supported the concept of a transgenic overexpression of Norrin in the sensory retina with preference to its inner layers.

Since the Pax6 promoter element that was used to drive the expression of transgenic Norrin becomes active at around embryonic (E) day 10.5^32^, we performed a detailed phenotype analysis to exclude any effects of transgenic Norrin on ocular development. In 6-week-old adult animals, no obvious differences were observed with regards to eye size, retinal structure, total retinal thickness, and thicknesses of outer and inner nuclear layer between Pax6-Norrin mice and their wild-type littermates (Supplemental Fig. 1). In addition, no differences in structure and number of optic nerve axons, or in structure of chamber angle and trabecular meshwork outflow pathways were observed between six-week-old Pax6-Norrin mice and wild-type controls (Supplemental Figs 2 and 3). Overall, our data strongly indicated that under physiological conditions a moderate transgenic overexpression of Norrin has no obvious influences on ocular morphogenesis and maintenance.

We next bred Pax6-Norrin mice for seven generations into the DBA/2J genetic background. Again we analyzed if the transgenic expression of Norrin causes obvious developmental changes in the optic nerve and its axons in the DBA/2J genetic background, and found no obvious structural differences in the morphology of retinae and optic nerves, or the number of optic nerve axons between 6-week-old DBA/2J/Pax6-Norrin mice and wild-type littermates (Supplemental Fig. 4). Further on, by collagen type IV staining of the basal lamina of microvascular endothelial cells to identify retinal vasculature^34^ no obvious changes between ten-month-old transgenic DBA/2J and wild-type littermates were observed (Supplemental Fig. 5). In three-week-old DBA/2J mice only a faint cytoplasmic and nuclear localization of β-catenin in the RGC layer was detected in DBA/2J mice by immunohistochemistry, whereas in transgenic mice the signal for β-catenin was considerably stronger (Fig. 1E) indicating that transgenic Norrin expression enhances Wnt/β-catenin signaling in inner retina of DBA/2J/Pax6-Norrin mice.

### Norrin overexpression attenuates glaucomatous optic nerve axonal damage

**Figure 2:**
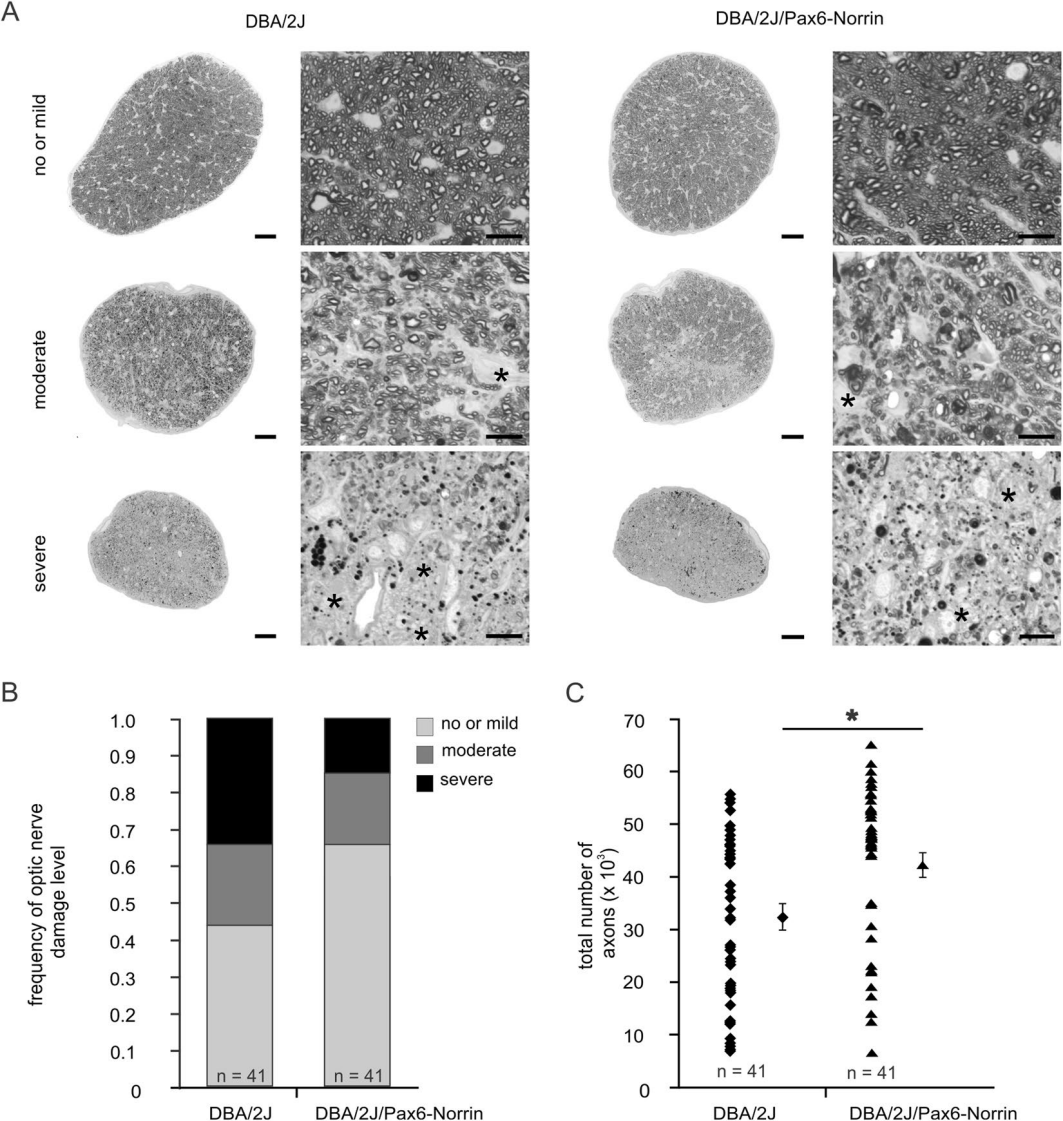
Transgenic Norrin protects retinal ganglion cell axons against glaucomatous degeneration. (**A**) Light microscopy of optic nerve cross sections (scale bar. 50 µm) and higher magnifications (scale bar. 10 µm) from one-year-old DBA/2J mice and DBA/2J/Pax6-Norrin littermates with mild or no, moderate and severe damage. (**B**) Semiquantitative analysis of mild or no, moderate and severe damage plotted as percentage. (**C**) Quantitative analysis of optic nerve axon number (mean ± SEM; *p < 0.01).

We next investigated glaucomatous optic nerve damage in one-year-old DBA/2J/Pax6-Norrin mice and their DBA/2J littermates. For semiquantitative evaluation of damage we used a grading scheme introduced previously for DBA/2J mice by John and colleagues^24^. According to this scheme, no or mild glaucomatous damage is present when the estimated decrease in nerve diameter is no greater than 30% compared to a typical normal nerve. Damage is moderate when the estimated decrease in nerve diameter is 30-60% compared to a typical normal nerve and severe if it is greater than 60%. Applying this scheme in our study, 34.1% of optic nerves from one-year-old DBA/2J mice had severe damage, while 22.0% and 43.9% had moderate or mild damage, respectively (Fig. 2A,B). In contrast, 85.3% of optic nerves from transgenic DBA/2J/Pax6-Norrin littermates had no or mild damage (Fig. 2A,B). The semiquantitative results were confirmed by a quantitative evaluation of optic nerve axons. The average number of remaining axons in one-year-old DBA/2J mice was 32,679 ± 2,429 (mean ± SEM) while on average approximately 30% more axons were detected in DBA/2J/Pax6-Norrin (42,461 ± 2,243; Fig. 2C), a difference that was statistically significant (p < 0.01; n = 41). Severity specific comparison showed a significantly increased number of axons in DBA/2J/Pax6-Norrin mice (51,186 ± 1,053; p < 0.05) compared to DBA/2J littermates (46,058 ± 2,034) in optic nerves that were classified as normal. However, no differences in the axon number between transgenic DBA/2J and wild-type littermates were observed in moderate and severe damaged optic nerves. Moreover, in one-year-old transgenic Pax6-Norrin mice the cell number in the RGC layer was significantly increased by more than 20% (55.3 ± 2.3 cells per 1,000 µm retina, n = 23, p < 0.01) compared to wild-type DBA/2J littermates (44.9 ± 2.9 per 1,000 µm retina, n = 28), confirming our observation of more axons in optic nerves from DBA/2J/Pax6-Norrin mice. Recordings of electroretinograms (ERG) showed a higher amplitude of the b-wave under scotopic conditions in six-month-old DBA/2J/Pax6-Norrin mice compared to DBA/2J littermates (Supplemental Fig. 6A,C), an observation that is consistent with a reduced degeneration in the inner retina of transgenic mice. However, no obvious differences between six-month-old DBA/2J/Pax6-Norrin mice and DBA/2J littermates regarding their amplitude of the a- and b-wave were detected under photopic conditions (Supplemental Fig. 6D,F). Moreover, in transgenic DBA/2J mice the implicit time of the b-wave was shorter compared to that of DBA/2J controls under scotopic and photopic conditions (Supplemental Fig. 6A,B,E), suggesting that the transgenic expression of Norrin can maintain inner retinal function of DBA/2J mice.

### The protective effects of Norrin are associated with increased retinal Igf1 expression and PI3K-Akt signaling

**Figure 3:**
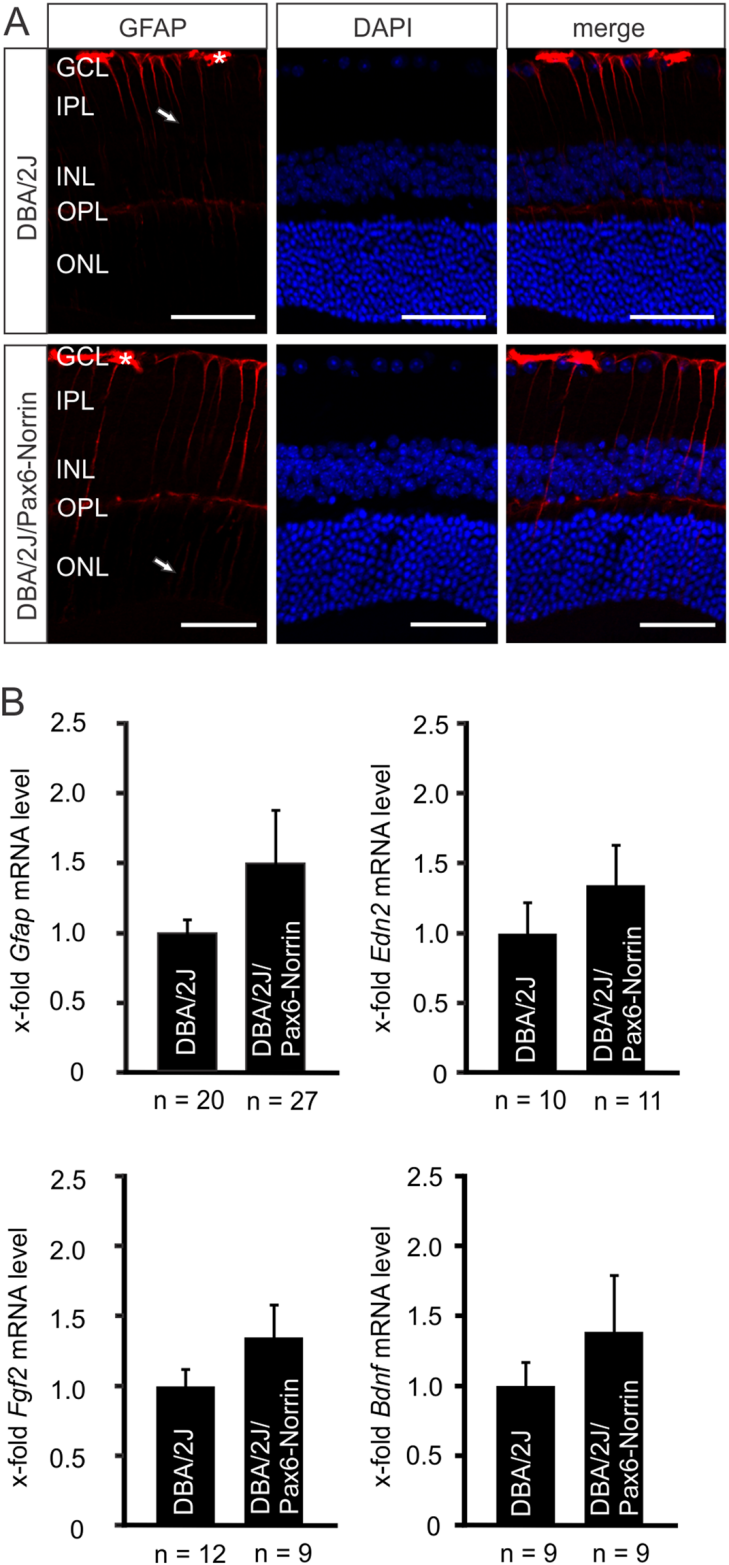
Norrin attenuates glaucomatous axonal damage without enhanced expression of *Gfap*, *Edn2*, *Fgf2* or *Bdnf*. (**A**) Immunohistochemistry for GFAP (red) in retinae of two-month-old DBA/2J/Pax6-Norrin mice and DBA/2 littermates. In DBA/2J mice GFAP staining is seen in astrocytes and Müller cell endfeet in the RGC layer and in radial Müller cell processes that extend into the inner plexiform layer. In DBA/2J/Pax6-Norrin littermates the radial staining is more pronounced and reaches into the inner nuclear layer. GCL, ganglion cell layer; IPL, inner plexiform layer; INL, inner nuclear layer; OPL, outer plexiform layer; ONL, outer nuclear layer. Scale bar. 50 µm. Nuclei are labeled with DAPI (blue). (**B**) Real-time RT-PCR for *Gfap*, *Edn2*, *Fgf2* and *Bdnf* in RNA from retinae of eight-week-old DBA/2J/Pax6-Norrin mice and DBA/2J littermates (mean ± SEM).

To investigate if the constitutive transgenic overexpression of Norrin in DBA/2J mice causes Müller glia reactivity, which can lead to an induction of protective factors, we analyzed the localization of glial fibrillary acidic protein (GFAP), a marker for astrocyte and Müller cell reactivity, in retinae of two-month-old mice before the onset of glaucomatous damage. By immunohistochemistry, a signal for GFAP was seen in the RGC layer of DBA/2J mice, a localization that corresponds with that of retinal astrocytes and Müller cell endfeet, and a Müller cell-characteristic radial staining was observed that extended from the RGC layer into the inner plexiform layer (Fig. 3A). In DBA/2J/Pax6-Norrin mice the radial staining was more pronounced and reached into the inner nuclear layer (Fig. 3A), suggesting increased Müller cell reactivity in this mouse strain. Quantitative real time RT-PCR showed a trend towards a higher *Gfap* expression in transgenic DBA/2J/Pax6-Norrin mice than in DBA/2J littermates, which was not significant (Fig. 3B). In addition, there were no significant differences in the retinal expression of *Edn2*, *Fgf2* and *Bdnf* between DBA/2J/Pax6-Norrin mice and their DBA/2J littermates (Fig. 3B). In both mouse strains, retinal expression of *Lif* was not detectable.

**Figure 4:**
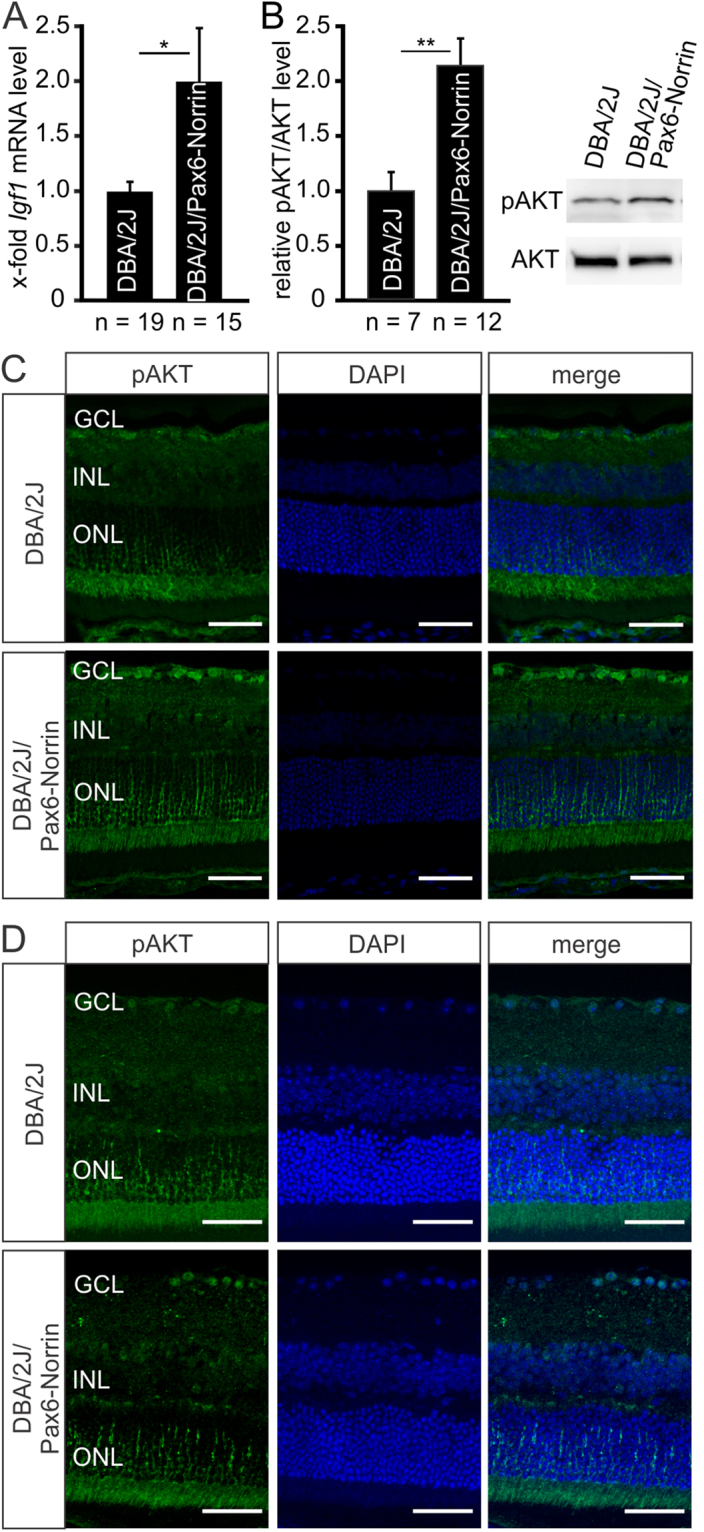
Norrin promotes activation of PI3K-Akt signaling via induction of IGF-1. (**A**) Real-time RT-PCR for *Igf1* in retinal RNA of eight-week-old DBA/2J/Pax6-Norrin mice and DBA/2J littermates (mean ± SEM; *p < 0.05). (**B**) Western blot analysis and relative densitometry for pAKT and AKT in retinal proteins from two-month-old DBA/2J/Pax6-Norrin mice and DBA/2J littermates. For relative densitometry the pAKT/AKT ratio was determined and set to 1 in DBA/2J mice (mean ± SEM; **p < 0.01). (**C**,**D**) Immunohistochemistry for pAKT (green) in retinae from DBA/2J and DBA/2J/Pax6-Norrin mice at the age of two (**C**) and ten (**D**) months. In both strains immunoreactivity for pAKT is observed in the outer nuclear layer. In DBA/2J mice pAKT immunoreactivity is weak in the ganglion cell layer but intense in two-month-old (**C**) and a little less pronounced in ten-month-old (**D**) DBA/2J/Pax6-Norrin littermates. Nuclei are labeled with DAPI (blue). GCL. ganglion cell layer; INL. inner nuclear layer; ONL. outer nuclear layer. Scale bars: 50 µm.

As we had previously shown an increase in retinal IGF-1 and its mRNA in another mouse model (βB1-Norrin) with Norrin overexpression^12^, we investigated the retinal IGF-1 mRNA levels in DBA/2J/Pax6-Norrin mice and found them to be twice as high (p < 0.05) as those in DBA/2J littermates (Fig. 4A). IGF-1 has neuroprotective properties that are mediated through PI3K/Akt signaling^35–37^. By immunohistochemistry of retinae from two-month-old mice, a specific signal for phosphorylated AKT (pAKT) was observed in the outer nuclear layer of DBA/2J mice, whereas pAKT immunoreactivity was weak in the ganglion cell layer (Fig. 4C). In marked contrast, in DBA/2J/Pax6-Norrin animals a distinct cellular immunostaining for pAKT was found in the ganglion cell layer (Fig. 4C). An enhanced immunoreactivity for pAKT was also seen in RGC of ten-month-old transgenic mice, even it was less pronounced when compared to two-month-old animals (Fig. 4D). When performing western blot analyses for pAKT and Akt in retinal proteins from two-month-old DBA/2 mice and their DBA/2J/Pax6-Norrin littermates, we detected distinct bands (Fig. 4B). Relative densitometry showed that the pAKT/AKT ratio was twice as high (p < 0.01) in DBA/2J/Pax6-Norrin mice indicating a substantial activation of PI3K/Akt signaling (Fig. 4B).

### The role of intraocular pressure and microglia reactivity in DBA/2J/Pax6-Norrin glaucoma

**Figure 5:**
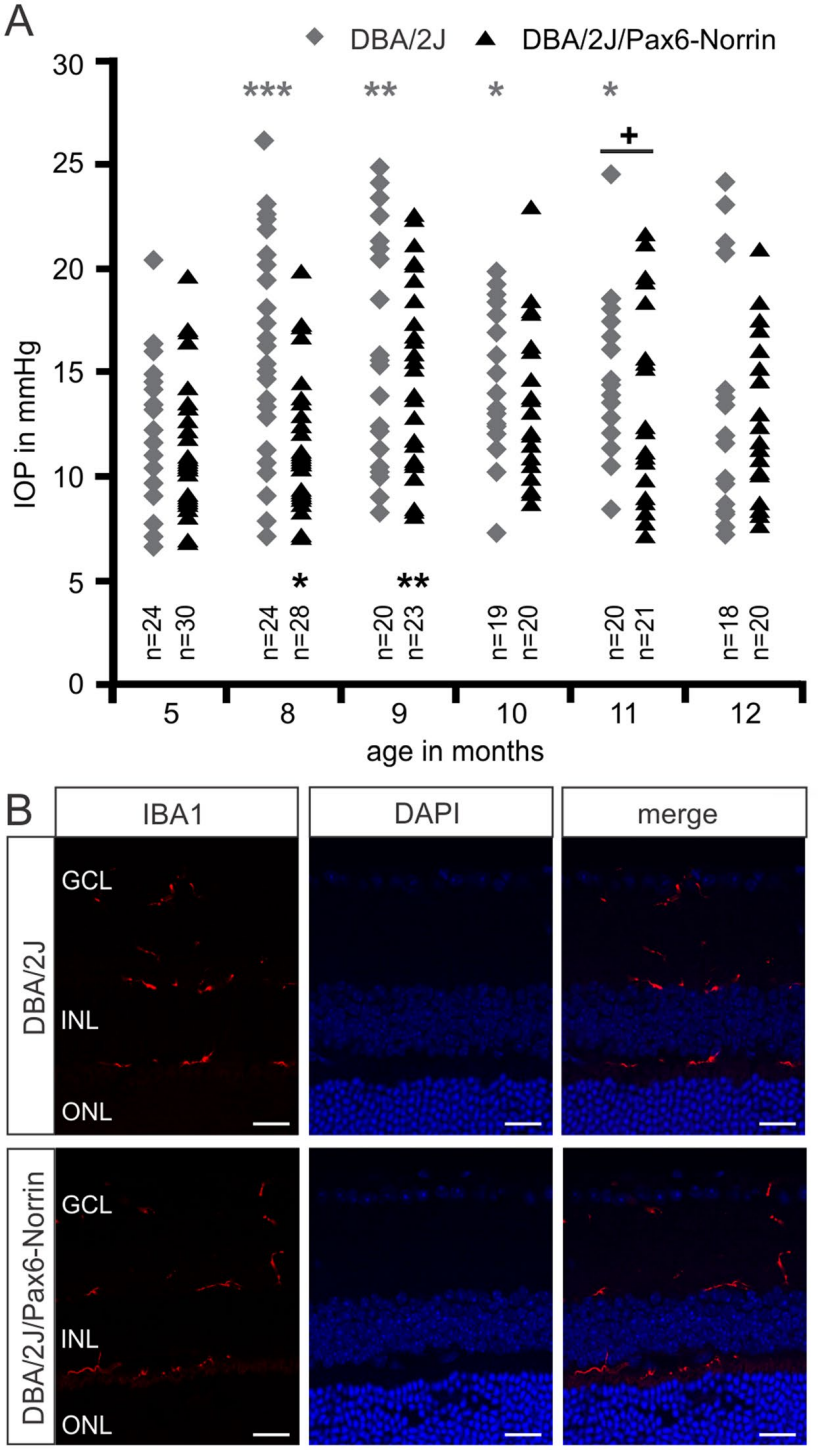
Intraocular pressure and retinal microglia reactivity in DBA/2J/Pax6-Norrin glaucoma. (**A**) Intraocular pressure (IOP) of transgenic DBA/2J/Pax6-Norrin and DBA/2J wild-type littermates (WT) was measured at the age of 5 to 12 months (mean ± SEM; n ≥ 18). For statistical analysis a one-way ANOVA between IOP from transgenic and wild-type mice (+) as well as between IOP of several months and their five months baseline levels (*) was performed (^+^p < 0.05; *p < 0.05; **p < 0.01; ***p < 0.001). (**B**) Immunoreactivity for IBA1 (red) in retinae of eight-week-old DBA/2J and DBA/2J/Pax6-Norrin littermates. Nuclei are stained with DAPI (blue). GCL. ganglion cell layer; INL. inner nuclear layer; ONL. outer nuclear layer; Scale bars: 20 µm.

Glaucoma in DBA/2J mice is associated with an increase in IOP that characteristically rises in the second half of the first year of life and returns to levels seen in young animals at around one year of age^24,25,38^. In addition, there is a substantial neuroimmunological component involving retinal microglia reactivity and invasion of macrophages into the optic nerve head^39,40^. We therefore analyzed if a substantial attenuated IOP or microglia reactivity contribute to the protective effects on RGC axons. During their first postnatal year DBA/2J/Pax6-Norrin mice and their DBA/2J littermates showed essentially the same development of IOP as reported by others (Fig. 5A). In five-month-old DBA/2J/Pax6-Norrin and DBA/2J mice, IOP was 12.2 ± 0.7 and 12.0 ± 0.6 mmHg, respectively. In the following months, IOP of both mouse strains increased and was significantly elevated at 8 (DBA/2J mice, 16.5 ± 1.2 mmHg; DBA/2J/Pax6-Norrin, 14.7 ± 1.0 mmHg), 9 (DBA/2J mice, 16.3 ± 1.2 mmHg; DBA/2J/Pax6-Norrin, 15.1 ± 0.9 mmHg) and 10 months (DBA/2J mice, 15.0 ± 0.8 mmHg; DBA/2J/Pax6-Norrin, 13.7 ± 0.9 mmHg) of age when compared to levels seen at 5 months. During those months, IOP in DBA/2J/Pax6-Norrin was not significantly different to that of DBA/2J animals. In 11-month-old DBA/2J/Pax6-Norrin mice, IOP had returned to levels seen at 5 months of age (12.6 ± 1.0 mmHg), while it was still significantly elevated in DBA/2J littermates and significantly higher (14.4 ± 0.6 mmHg; p < 0.05) than in DBA/2J/Pax6-Norrin mice. In both mouse strains, IOP at one year of age was not significantly different from that seen at 5 months of age (DBA/2J mice, 13.0 ± 1.3 mmHg; DBA/2J/Pax6-Norrin, 12.7 ± 0.8 mmHg). To assess the phenotype of microglia before onset of glaucomatous damage, retinae were immunostained with antibodies against IBA1. Ramified microglia cells were identified in the ganglion cell layer, at the inner and outer borders of the inner plexiform layer and within the innermost surface of the outer plexiform layer (Fig. 5B) essentially corroborating previous reports^39^. No obvious differences in shape and localization of microglial cells were seen when DBA/2J/Pax6-Norrin mice were compared with DBA/2J littermates (Fig. 5B).

### Anterior segment phenotype in DBA/2J/Pax6-Norrin glaucoma

**Figure 6:**
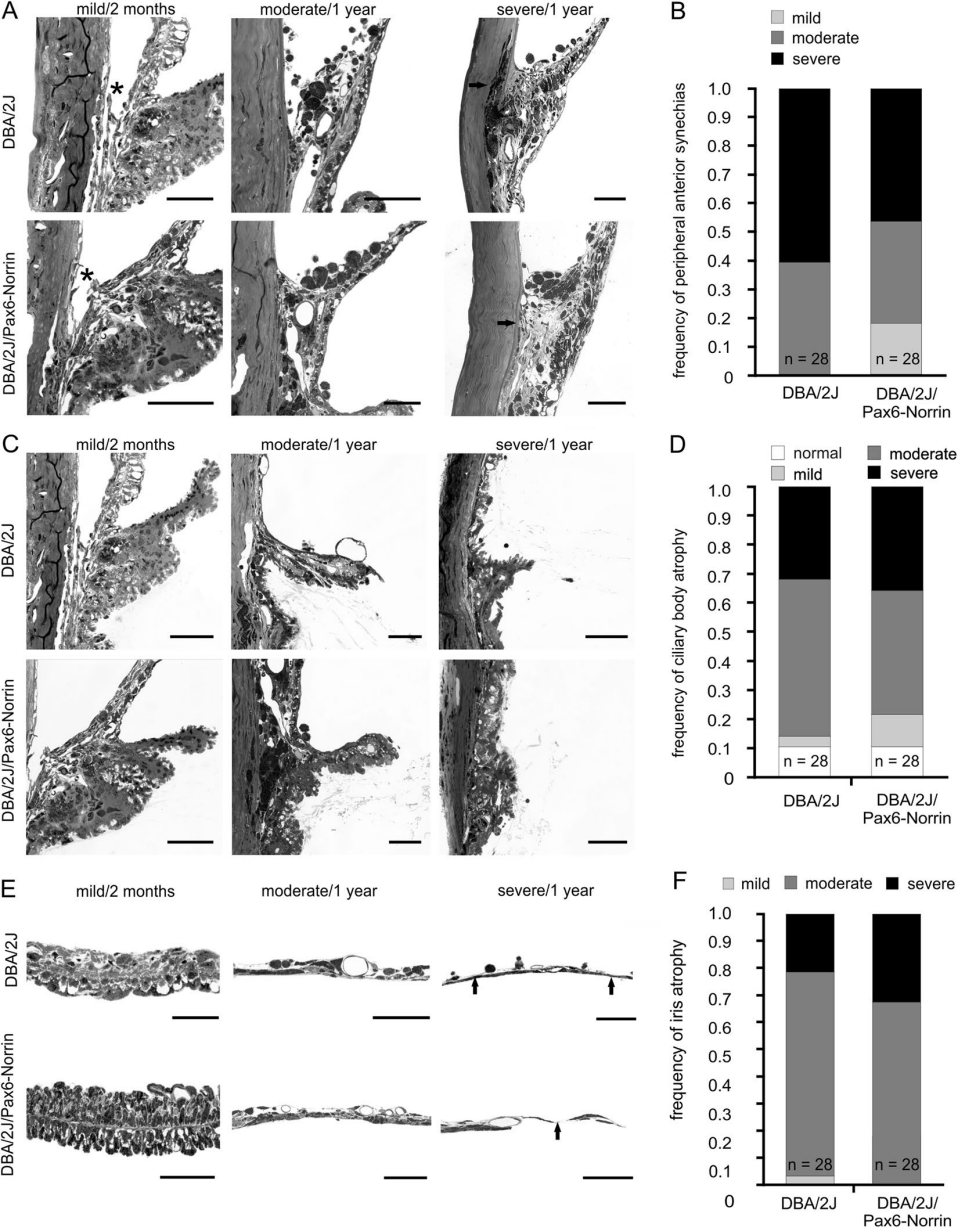
Anterior eye segment pathology in DBA/2J and DBA/2J/Pax6-Norrin littermates. (**A**,**C**,**E**) Light micrographs (Richardson’s stain; scale bars: 50 µm; asterisk, open chamber angle; arrows, atrophic iris) indicating mild, moderate and severe damage in chamber angle, ciliary body and iris at 2 months or 1 year of age. (**B**,**D**,**F**) Semiquantitative grading of changes plotted as percentage.

As we had observed lower IOP in 11-month-old DBA/2J/Pax6-Norrin mice than in their DBA/2J littermates, we analyzed the structural details of the anterior eye segment of both strains at one year of age to check for differences. For semiquantitative analysis of the pathological changes, we used a grading system published previously that identifies no or mild, moderate and severe changes^24^. In DBA/2J mice, 38% and 62% of eyes showed a moderate or severe occlusion of the chamber angle structure, respectively (Fig. 6A,B). In contrast, in eyes from DBA/2J/Pax6-Norrin mice severe damage was seen in 44% of the eyes (Fig. 6A,B). No or mild changes were seen in the chamber angle of 20% of DBA/2J/Pax6-Norrin eyes, but in no DBA/2J eye (Fig. 6A,B). Moreover, the degree of ciliary body and iris atrophy appeared to be rather similar between DBA/2J/Pax6-Norrin mice and their DBA/2J littermates (Fig. 6C–F).

### Transgenic overexpression of Norrin enhances Wnt/β-catenin signaling in the trabecular meshwork

**Figure 7:**
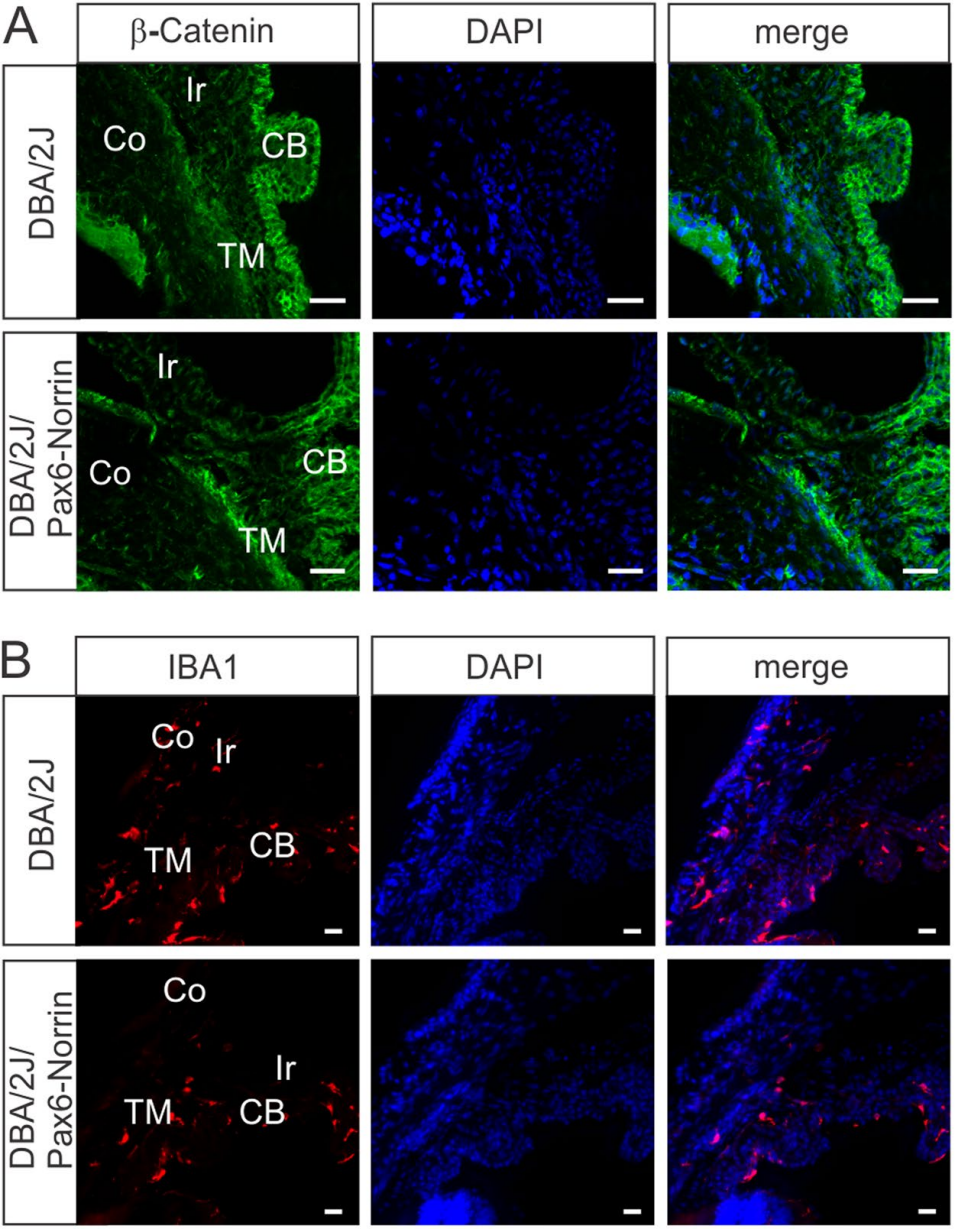
Transgenic overexpression of Norrin enhances Wnt/β-catenin signaling in the trabecular meshwork. (**A**) Immunostaining for β-catenin (green) in the anterior eye segment of two-month-old mice. In DBA/2J mice a marked signal for β-catenin is observed in the epithelium of the ciliary body (CB), whereas immunoreactivity in the trabecular meshwork (TM) is weak. In contrast, in the TM of DBA/2J/Pax6-Norrin mice intense staining for β-catenin is detected. Ir, iris; Co, cornea; blue, DAPI staining; scale bars: 20 µm. (**B**) In DBA/2J/Pax6-Norrin and their DBA/2J littermates IBA1 positive cells (red) are present in ciliary body (CB), iris (Ir) and trabecular meshwork (TM). Both strains show no differences in the distribution or number of IBA1 positive cells. Co, cornea; blue, DAPI staining; scale bars: 20 µm.

Since the semiquantitative grading of chamber angle pathology had indicated a somewhat less severe damage in DBA/2J/Pax6-Norrin mice, we wondered if this effect could be mediated by a Norrin-mediated enhancement of Wnt/β-catenin signaling. By immunohistochemistry, in two-month-old DBA/2J mice a marked signal for β-catenin was observed in the epithelium of the ciliary body, whereas immunoreactivity in the trabecular meshwork was weak (Fig. 7A). In contrast, in the trabecular meshwork of DBA/2J/Pax6-Norrin mice intense staining for β-catenin was detected (Fig. 7A) suggesting enhanced Wnt/β-catenin signaling. For DBA/2J mice an invasion of bone marrow-derived leukocytes^41^ and an enhanced activity of some components of the ocular immune system^42–44^ has been implicated to play a causative role in anterior eye segment pathology. To analyze, if a difference in the number of macrophages might play role in the Norrin-mediated effects on chamber angle pathology, we visualized macrophages by IBA1 immunostaining of anterior eye segments in two-month-old mice. In DBA/2J/Pax6-Norrin and their DBA/2J littermates we detected IBA1 positive cells in ciliary body, iris and trabecular meshwork. Both strains showed no differences in the distribution of IBA1 positive cells (Fig. 7B). The quantitative analysis of IBA1 positive cells in the trabecular meshwork showed an essentially similar number of cells in DBA/2J/Pax6-Norrin animals (0.82 ± 0.31 per 1,000 µm^2^; n = 8) and DBA/2J littermates (0.83 ± 0.28 per 1,000 µm^2^; n = 4). Even though we can not rule out any Norrin-mediated effects on IBA1 positive cells in older animals, our observations indicate that Norrin-mediated Wnt/β-catenin signaling has no influence on the number of IBA1 positive cells in the trabecular meshwork at disease onset.

## Discussion

We conclude that a constitutive increase in the retinal amounts of Norrin protects RGC and their axons from chronic glaucomatous damage, an effect that is mediated via the Wnt/β-catenin-induced increase in IGF-1 expression causing the enhanced activity of neuroprotective PI3K/Akt signaling. This conclusion rests upon (1) the observation that overexpression of Norrin in the retina leads to an accumulation of β-catenin in nuclei of inner retina neurons, (2) the finding that axonal damage in hereditary DBA/2J glaucoma is markedly attenuated in the presence of increased levels of Norrin and β-catenin, and (3) the high quantities of IGF-1 mRNA and of pAkt in glaucomatous retinae with elevated levels of Norrin and β-catenin.

In experimental models of acute neuronal damage in the retina, Norrin exerts its protective effects by augmenting endogenous protective signaling pathways that are quickly induced upon injury. Light damage of photoreceptors causes an increase in the retinal expression of both EDN2 and BDNF^18,45–48^, an effect that is enhanced when transgenic Norrin is overexpressed in the outer retina^18^. Excitotoxic acute RGC damage causes increased expression of EDN2, FGF2 and LIF, which are all further induced in the presence of recombinant Norrin^20^. LIF, EDN2, FGF2, or BDNF are components of intrinsic interconnected protective signaling networks that ameliorate photoreceptor or RGC damage after acute injury^2,20,49^. The signaling networks appear to be less relevant though for RGC protection during chronic glaucomatous damage in DBA/2J mice. LIF, which is highly expressed following acute photoreceptor or RGC injury^18,20,49^, was not detectable in the DBA/2J retina in the present study. Retinal EDN2 is induced early in DBA/2J glaucoma^50^, but quite in contrast to acute injury models it likely rather accelerates glaucoma damage than protecting from it. It is actually the treatment with an endothelin receptor antagonist that protects DBA/2J mice from axonal damage^50^. In any case, the findings of the present study clearly indicate that the protective effects of Norrin against chronic glaucomatous damage do not involve an increased expression of EDN2, FGF2, LIF or BDNF in the DBA/2J retina.

Similar to another mouse line with transgenic Norrin overexpression in the eye, DBA/2J/Pax6-Norrin mice show a robust induction of IGF-1 in their retina^12^. In a previous study, we observed that Norrin induces the expression of IGF-1 in both Müller cells and microvascular endothelial cells via the activation of canonical Wnt/β-catenin signaling^12^, and it appears reasonable to assume that the same holds true for DBA/2J/Pax6-Norrin mice. IGF-1 is well known for its neuroprotective properties that are mediated through PI3K/Akt signaling^35–37,51^. Consequently, IGF-1-induced high levels of pAKT are likely an essential component of Norrin’s protective effects against glaucomatous damage in DBA/2J/Pax6-Norrin mice. The site of optic nerve axonal damage in DBA/2J mice is at an astrocyte-rich region of the optic nerve just posterior to the retina (“glial lamina”)^28^. Axonal damage at the glial lamina is the primary event that subsequently leads to RGC apoptosis^28,29^. If apoptosis of the RGC soma is prevented, optic nerve axons continue to degenerate^28^. There is considerable evidence that the PI3K/Akt pathway is particularly important for mediating neuronal survival under a wide variety of circumstances^52,53^. Quite intriguingly, PI3K/Akt signaling acts both in the soma and the distal axon of neurons to prevent apoptosis^54^, a scenario that might be very relevant for its protective effects of optic nerve axons in DBA/2J glaucoma. In both DBA/2J and DBA/2J/Pax6-Norrin mice, we found axonal damage at the optic nerve head to be quite variable, corroborating findings of others^25^. Because of the high variability, we followed the recommendation by Libby and co-workers to use more than 40 animals per group when assessing differences in DBA/2J optic nerve axonal damage^25^. Quite intriguingly, by ERG recordings from DBA/2J/Pax6-Norrin mice we observed a shorter implicit time and higher amplitude of the b-wave, which are both indicators for a less severe degeneration in the inner retina^55^. From the age of 6 months on DBA/2J mice have a prolonged implicit time of the b-wave when compared to younger age^56^. Since low-grade inflammatory processes or other so far unknown mechanisms in the preclinical phase before IOP increase are accused to be involved in mediating glaucomatous damage of DBA/2J mice^57^, it is tempting to speculate that Norrin could influence these processes most likely via an enhanced AKT signaling.

To date, the DBA/2J mouse strain is the most thoroughly characterized mouse model of hereditary glaucoma^24–26,29^. The animals suffer from both iris pigment dispersion and iris stromal atrophy that are caused by mutations in the genes *Gpnmb* and *Tyrp1* ^44^. The mutations that are essential for glaucomatous damage in this mouse model^58^ appear to promote toxic events during melanogenesis resulting in leakage of toxic molecules from melanosomes^59^. Subsequently, an ocular inflammatory response is generated that further amplifies the pigment dispersion leading to an attached iris to the cornea which in turn causes partial or complete occlusion of the chamber angle. Chamber angle occlusion typically results in impairment of aqueous humor outflow and is the likely cause for the IOP elevation in DBA/2J mice, which is typically observed at around 6 months of age^25^. High IOP is thought to trigger the axonal degeneration at the optic nerve head that characteristically becomes manifest at around 1 year of age^25^. Reducing IOP in DBA/2J mice genetically, surgically, or through pharmaceutical therapy prevents significant optic nerve axonal damage^60–64^. Quite comparable to the results of others^25^, we detected high IOP in DBA/2J and DBA/2J/Pax6-Norrin mice during the second half of their first year of life, and assume that this increase triggered the axonal damage that we observed in one-year-old animals. We also observed the characteristic IOP decrease at the end of the first year, a scenario that is thought to be caused by the significant atrophy of the ciliary body at this age^25^. The fact that IOP decreased one month earlier in DBA/2J/Pax6-Norrin mice when compared to their DBA/2J littermates might indicate more pronounced ciliary body atrophy in DBA/2J/Pax6-Norrin mice, although we could not find evidence for this.

In addition to high IOP, microglia reactivity, monocyte entry into optic nerve and retina and complement activation are essential components of optic nerve axonal degeneration in DBA/2J mice, as their prevention protects from glaucomatous damage, even in the continuous presence of elevated IOP^40,65–68^. Secreted transgenic Norrin in DBA/2J/Pax6-Norrin mice may well act on the biology of retinal/optic nerve head macroglia, or invading monocytes contributing to its protective effects on glaucomatous axonal damage. At first glance though, we did not find obvious differences in macroglia or microglial reactivity between DBA/2J and DBA/2J/Pax6-Norrin mice.

While we did not observe differences in ciliary body or iris atrophy between one-year-old DBA/2J mice and their DBA/2J/Pax6-Norrin littermates, the number of animals with only mild or moderate changes in the chamber angle was higher in the DBA/2J/Pax6-Norrin group, in which the amounts of β-catenin in the chamber angle were also elevated. It is of interest to note that secreted frizzled-related protein-1 (sFRP-1), an antagonist of Wnt signaling, is overexpressed in human glaucomatous trabecular meshwork cells^69^. Moreover recombinant sFRP-1 decreases outflow facility in *ex vivo* perfusion-cultured human eyes and reduces the trabecular levels of β-catenin^69^. In line, the inhibition of Wnt/β-catenin signaling in human trabecular meshwork cells induces increased cellular stiffness^70^, which leads to increased IOP *in vivo*. Quite intriguingly, the activation of the Wnt/β-catenin pathway inhibits the expression of extracellular matrix components^71^, which accumulate in the trabecular meshwork of glaucomatous eyes. Based on these studies, which all argue for a crucial role of Wnt/β-catenin signaling in the pathogenesis of glaucoma, it is tempting to speculate that a comparable scenario in DBA/2J/Pax6-Norrin mice might involve the Norrin-induced activity of canonical Wnt/β-catenin signaling attenuating the continuous structural changes in the chamber angle of DBA/2J/Pax6-Norrin mice, albeit not to an extent that significantly interferes with IOP elevation.

## Conclusions

Overall we identified important components of a protective signaling network that is powerful to attenuate optic nerve degeneration in chronic glaucoma. Norrin, canonical Wnt/β-catenin signaling, IGF-1 and enhanced activity of PI3K-Akt signaling are involved, each of which might be a promising target to further develop therapeutic strategies to prevent glaucomatous damage in human patients.

## Material and Methods

### Mice

All procedures conformed to the tenets of the National Institutes of Health Guidelines on the Care and Use of Animals in Research, the EU Directive 2010/63/E and the ARVO Statement for the Use of Animals in Ophthalmic and Vision Research, and were approved by the local authorities (Regierung der Oberpfalz, Bavaria, Germany). All experiments were performed in mice of either sex.

For generation of *Pax6-Norrin* mice, the *Pax6* fusion promoter containing both the *Acc*I fragment of the α-enhancer and the P0 minimal promoter fragment of *Pax6* was used^31^, and was cloned upstream of the murine Norrin cDNA (Fig. 1A; for detailed information see Supplemental Methods). Potential Pax6-Norrin transgenic mice were screened by PCR analyses using the primers 5′-GTGAAGGAACCTTACTTCTGTGGTG-3′ and 5′-GTCCTTGGGGTCTTCTACCTTTCTC-3 that amplify a 300 bp DNA fragment by using the thermal cycle profile of denaturation at 94 °C for 30 s, annealing at 55 °C for 30 s, and extension at 72 °C for 45 s for 30 cycles. To obtain Pax6-Norrin mice in the DBA/2J genetic background, transgenic mice were bred seven generations with DBA/2J wild-type mice (Charles River).

Electroretinograms were recorded binocularly in 6-month-old DBA/2J/Pax6-Norrin and DBA/2J littermates as described previously^10^. For detailed information see Supplemental Methods. IOP measurements were made using a rebound tonometer (TonoLAB) as described previously^72^. In brief, all measurements were conducted at the same time of day. After anesthesia with a mixture of ketamine (WDT) and xylazine (Serumwerk), six automatically averaged measurements were taken and indicated as one reading. A mean of five readings was considered as a single result. Highly and moderately variable readings were excluded.

### Microscopy

After enucleation, eyes and optic nerves were washed overnight in cacodylate buffer, post-fixed with OsO_4_, dehydrated, and embedded in Epon. One micrometer meridional semithin sections of eyes, cutting both optic nerve head and pupil were stained after Richardson’s^73^ whereas cross sections of optic nerves were stained with paraphenylenediamine^74^ and analyzed by light microscopy using an Axiovision microscope (Carl Zeiss). For calculation of the cell number in the RGC layer the number of nuclei in the RGC layer was quantified and correlated to 1000 µm of inner retinal surface. Quantification of axonal number in the optic nerve was performed following a protocol published previously^57^. In brief, 1 central and 4 peripheral quadrates (40 µm × 40 µm each), which cover more than 10% of total optic nerve area, were projected onto optic nerves, quantified and extrapolated to the total area of the optic nerve. For each group (DBA/2J and DBA/2J/Pax6-Norrin) 41 optic nerves from 46 animals of either sex were evaluated. For semiquantitative analyses of peripheral anterior synechiae, iris pigment epithelium atrophy, ciliary body atrophy and optic nerve atrophy, lesions were graded in no or mild as well as normal, moderate and severe as described previously for the analysis of structural changes in DBA2J mice by John and colleagues (see supplemental experimental procedures)^24^. To evaluate integrity and thickness of retinae, meridional retinal semithin sections were analyzed as described previously^18,75^. In brief, the distance between *ora serrata* and optic nerve head was divided into tenths, and thickness of the total retina, of the inner and outer nuclear layer was measured between each tenth using the Axiovision software 4.8 (Carl Zeiss). For immunohistochemistry, eyes from two-month-old mice were fixed for 4 h in 4% paraformaldehyde. After blocking, rabbit anti-glial fibrillary acidic protein (GFAP) antibodies (1:1,000; Dako), rabbit anti-β-catenin antibodies (1:100; Cell Signaling Technology), rabbit anti-pAKT antibodies (1:100; Abcam), rabbit anti-collagen type IV antibodies (1:100; Chemicon) and rabbit anti-ionized calcium-binding adapter molecule 1 (IBA1) antibodies (1:500; Wako Chemicals) were incubated overnight. After washing with PBS, for visualization of GFAP and IBA1 samples were treated for 1 h with Cy3 fluorescein-labeled goat-anti-rabbit antibodies (1:2,000; Dako), and of β-catenin, collagen type IV and pAKT with biotinylated goat anti-rabbit antibodies (1:500; Vector Laboratories) for 1 h, followed by a final incubation with Alexa 488-labeled streptavidin (1:1,000; Life Technologies). Sections were washed again and mounted in fluorescent mounting medium containing 1:10 DAPI (Sigma Aldrich), and analyzed on an Axio Imager fluorescence microscope with an integrated Apotome module (Carl Zeiss). For quantification of IBA positive cells in the trabecular meshwork, the number of cells was determined and correlated to the area of the trabecular meshwork in 8 sagittal sections of one eye.

### RNA analyses

Total RNA from mouse retinae was extracted using TriFast™ reagent (Peqlab) according to manufacturer’s instructions. cDNA was synthetized from total RNA using the iScript cDNA Synthesis Kit (BioRad). Quantitative real-time RT-PCR analyses were performed using the BioRad iQ5 Real-Time PCR Detection System. PCR reaction was performed in a volume of 15 µl, consisting of 1.5 µl of 10x PCR buffer, 0.6 µl of MgCl_2_ (25 mM), 0.12 µl of dNTPs (25 mM each; Qiagen), 0.6 µl of *Taq* (5 U/µL; Hot Star; Qiagen), 5 µl of primer mix (1 µM each, Life Technologies, Supplemental Table 1), and 0.39 µl of 1x SYBR green I solution (Sigma-Aldrich). The following temperature profile was used: 40 cycles of 10 s denaturation at 95 °C, 40 s of annealing and extension at 60 °C. For normalization, mRNA levels were compared to *Gnb2l*. All PCR primers were designed to span exon-intron boundaries and were purchased from Invitrogen (Table 1; Thermo Fisher). Northern blot analysis was performed as described previously, a detailed protocol is provided in supplemental experimental procedures^10^.

**Table 1:** Primers used for real-time RT-PCR.

gene/allele	forward	reverse
*Bdnf*	5′-AGTCTCCAGGACAGCAAAGC-3′	5′-TGCAACCGAAGTATGAAATAACC-3′
*Edn2*	5′-ACCTCCTCCGAAAGCTGAG-3′	5′-TTTCTTGTCACCTCTGGCTGTA-3′
*Fgf2*	5′-CGGCTCTACTGCAAGAACG-3′	5′-TGCTTGGAGTTGTAGTTTGACG-3′
*Gfap*	5′-ACAGACTTTCTCCAACCTCCAG-3′	5′-CCTTCTGACACGGATTTGGT-3′
*Gnb2l*	5′-TCTGCAAGTACACGGTCCAG-3′	5′-ACGATGATAGGGTTGCTGCT-3′
*Igf1*	5′-CAAAAGCAGCCCGCTCTA-3′	5′-TCGATAGGGACGGGGACT-3′
*Lif*	5′-AAACGGCCTGCATCTAAGG-3′	5′-AGCAGCAGTAAGGGCACAAT-3′

### Protein preparation and western blot analyses

Retinal proteins were isolated using TriFast™ reagent (Peqlab) according to manufacturer’s instructions. 25 µg of protein were subjected to a 10% SDS-PAGE and transferred on a PVDF membrane (Roche) by semidry blotting. After blocking with 5% BSA in TBS-T, membranes were incubated overnight at 4 °C with rabbit anti-pAKT (1:1,000 in 5% BSA/TBS-T; Abcam) or rabbit anti-β-catenin (1:1,000 in 5% BSA/TBS-T; Cell Signaling) antibodies, followed by an additional hybridization for 1 h with a HRP-conjugated goat anti-rabbit antibodies (1:2,000; Cell Signaling Technology). For visualization, membranes were incubated in Immobilon™ Western HRP substrate (Millipore) and visualized on a BAS 3000 Imager work station (Fujifilm). As loading control, for pAKT rabbit anti-AKT antibodies (1:1,000 in 5% BSA/TBS-T; Abcam) and for β-catenin, a HRP-conjugated anti-GAPDH antibodies (1:5,000 in 5% BSA/TBS-T; Rockland) were used, and evaluated by densitometry with the Aida Image Analyzer v.4.06 software (Raytest).

### Statistics

All results are expressed as mean ± SEM. Comparisons between the mean variables of 2 groups were made by a 2-tailed Student’s t-test. A one-way ANOVA was performed to compare the mean variables of more than 2 groups, followed by a LSD or Games Howell post-hoc test for data that meet or not meet the criteria of the assumption of homogeneity of variances, respectively. P-values less than 0.05 were considered to be statistically significant.

## Electronic supplementary material

Supplementary Information
