# Supplementary material for: Norrin protects optic nerve axons from degeneration in a mouse model of glaucoma
Source: Sci Rep. 2017 Oct 27;7:14274. doi: 10.1038/s41598-017-14423-8 (PMC5660254; doi:10.1038/s41598-017-14423-8)
Supplement: Supplementary file 1 — Supplementary Information [file 41598_2017_14423_MOESM1_ESM.pdf]

Norrin protects optic nerve axons from degeneration in a mouse model of glaucoma

Stephanie A. Leopold, Ludwig F. Zeilbeck, Gregor Weber, Roswitha Seitz, Michael R. Bösl, Herbert Jägle, Rudolf Fuchshofer, Ernst R. Tamm and Andreas Ohlmann

Supplemental Figures

Supplemental Figure 1

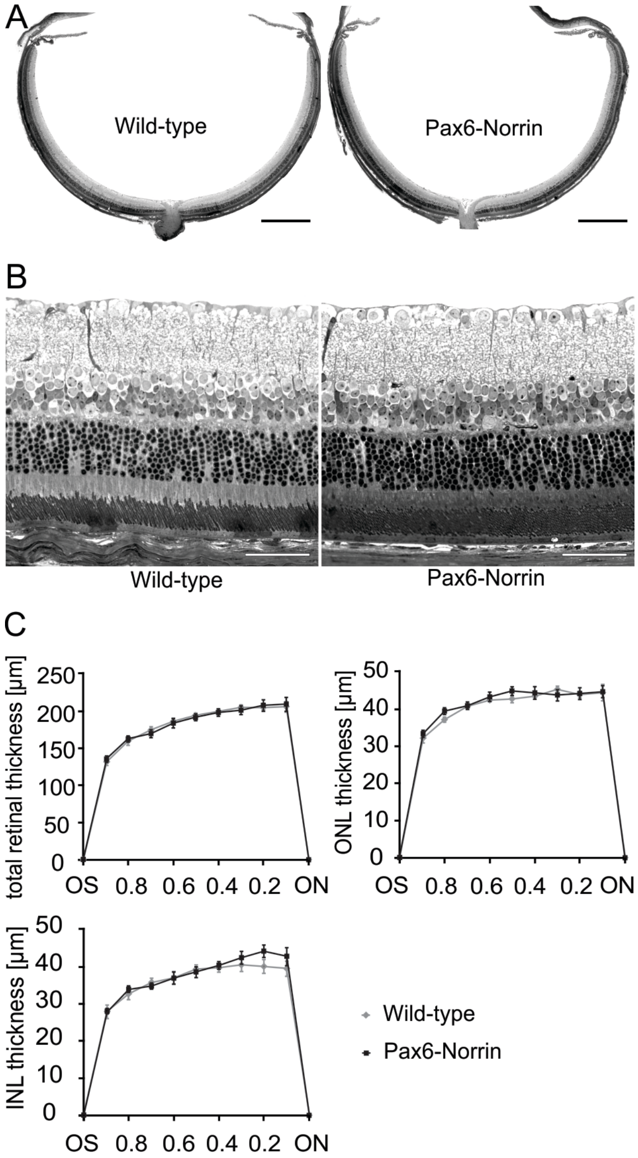

Supplemental Figure 1. Pax6-Norrin mice have no obvious retinal phenotype in the FVB/N/CD-1 genetic background. A, B. Light microscopy of meridional sections of the posterior globe (A) and higher magnifications of retinae (B) from transgenic six-week-old Pax6-Norrin mice and FVB/N/CD-1 wild-type littermates. Scale bars: A. 500 µm; B. 50 µm. **C.** Quantification of total retinal, inner nuclear layer (INL) and outer nuclear layer thickness (ONL). The distance between *ora serrata* (OS) and optic nerve head (ON) was divided into tenths, and thickness was measured between each tenth. The mean of each reading point was plotted in a spider diagram (mean ± SEM; n = 5).

Supplemental Figure 2

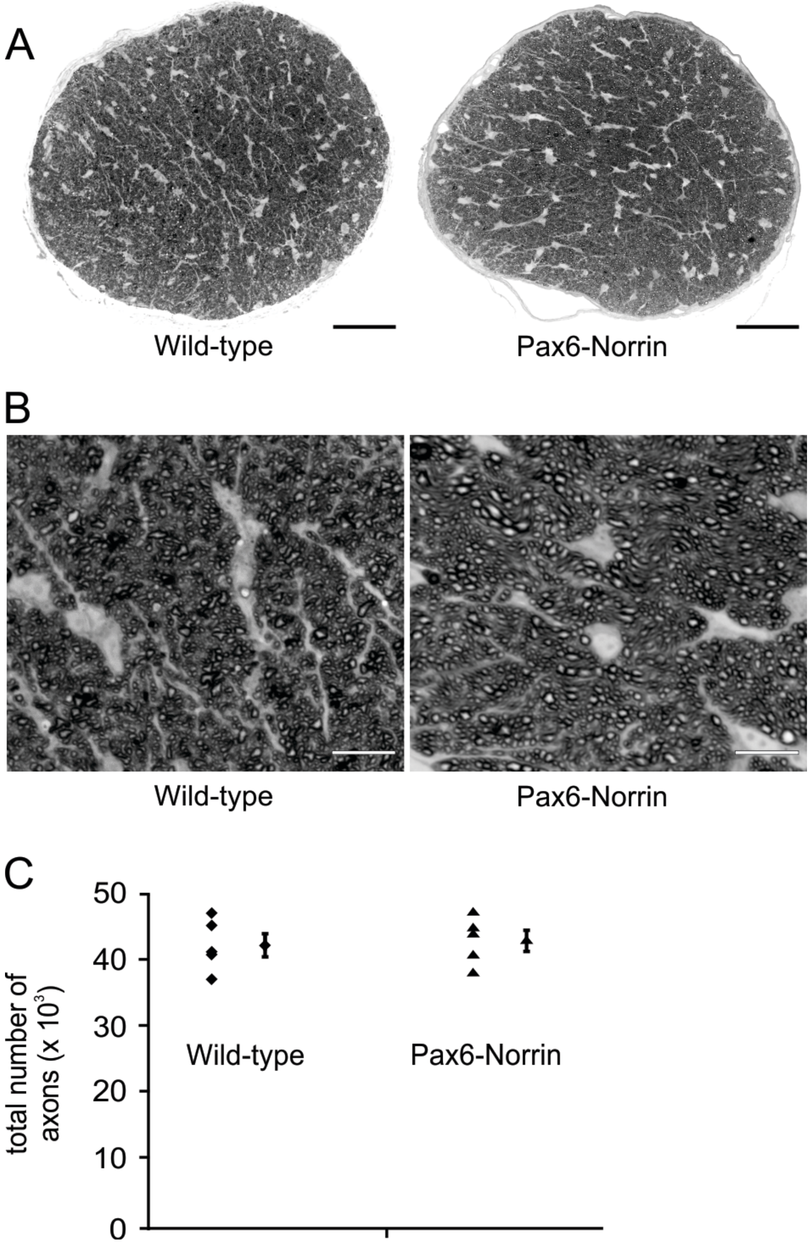

Supplemental Figure 2. Pax6-Norrin mice in the FVB/N/CD-1 genetic background have no obvious optic nerve phenotype or changes in the number of optic nerve axons. A, B. Light microscopy of optic nerve cross sections (A) and higher magnifications (B) of transgenic six-week-old Pax6-Norrin mice and FVB/N/CD-1 wild-type littermates. Scale bars: A. 50 µm; B. 10 µm. **C.** For quantification, the number of axons in optic nerves from Pax6-Norrin and wild-type littermates was quantified and plotted as total number of axons per optic nerve (mean ± SEM; n = 5).

Supplemental Figure 3

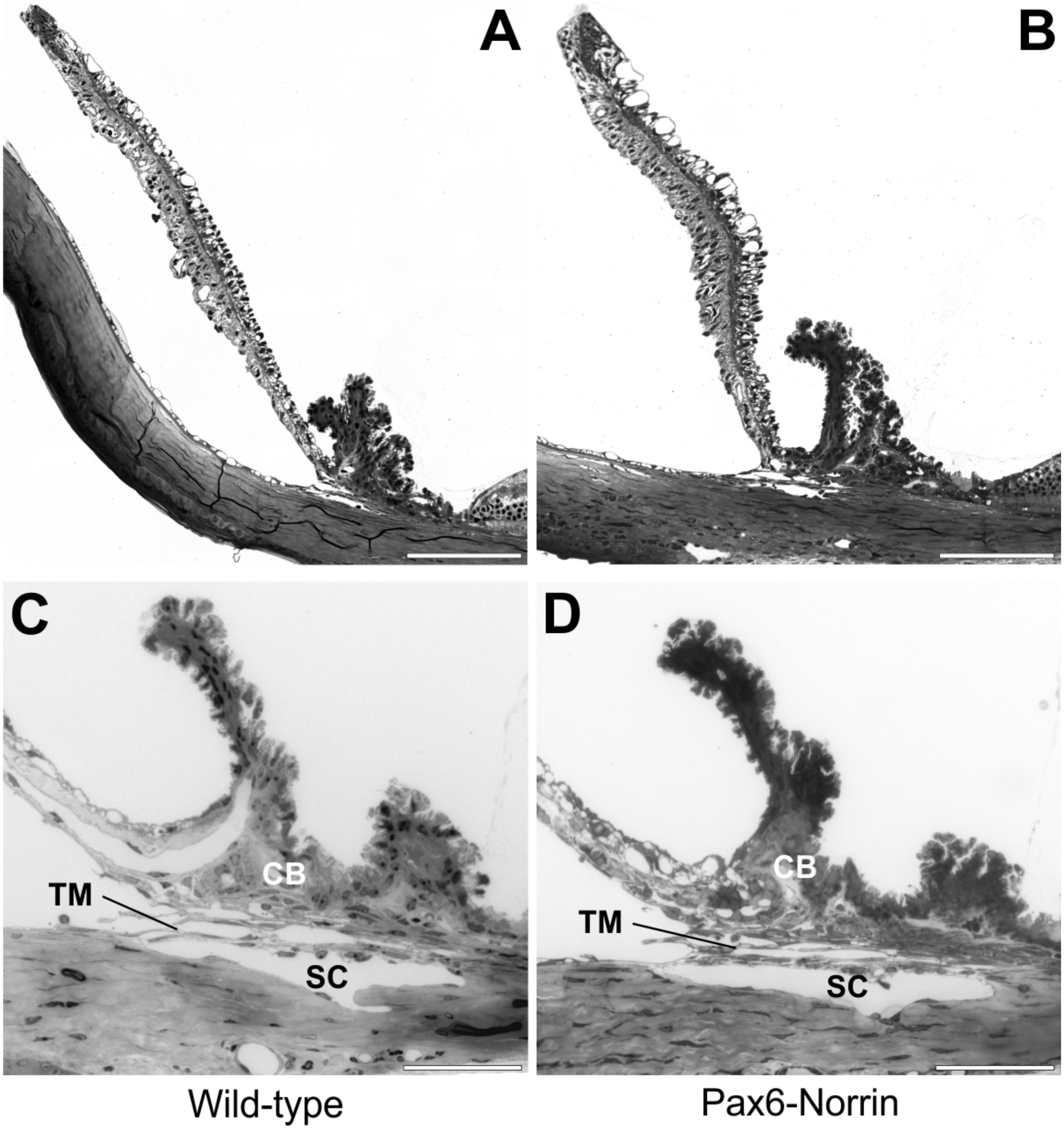

Supplemental Figure 3. Pax6-Norrin mice have no obvious anterior eye segment phenotype in the FVB/N/CD-1 genetic background. Representative light microscopy of the anterior eye segment (A, B) and the chamber angle (C, D) of transgenic six-week-old Pax6-Norrin mice and FVB/N/CD-1 wild-type littermates. Richardson's stain, scale bars: A, B. 100 µm; C, D. 20 µm; TM, trabecular meshwork; SC, Schlemm's canal; CB, cilary body.

Supplemental Figure 4

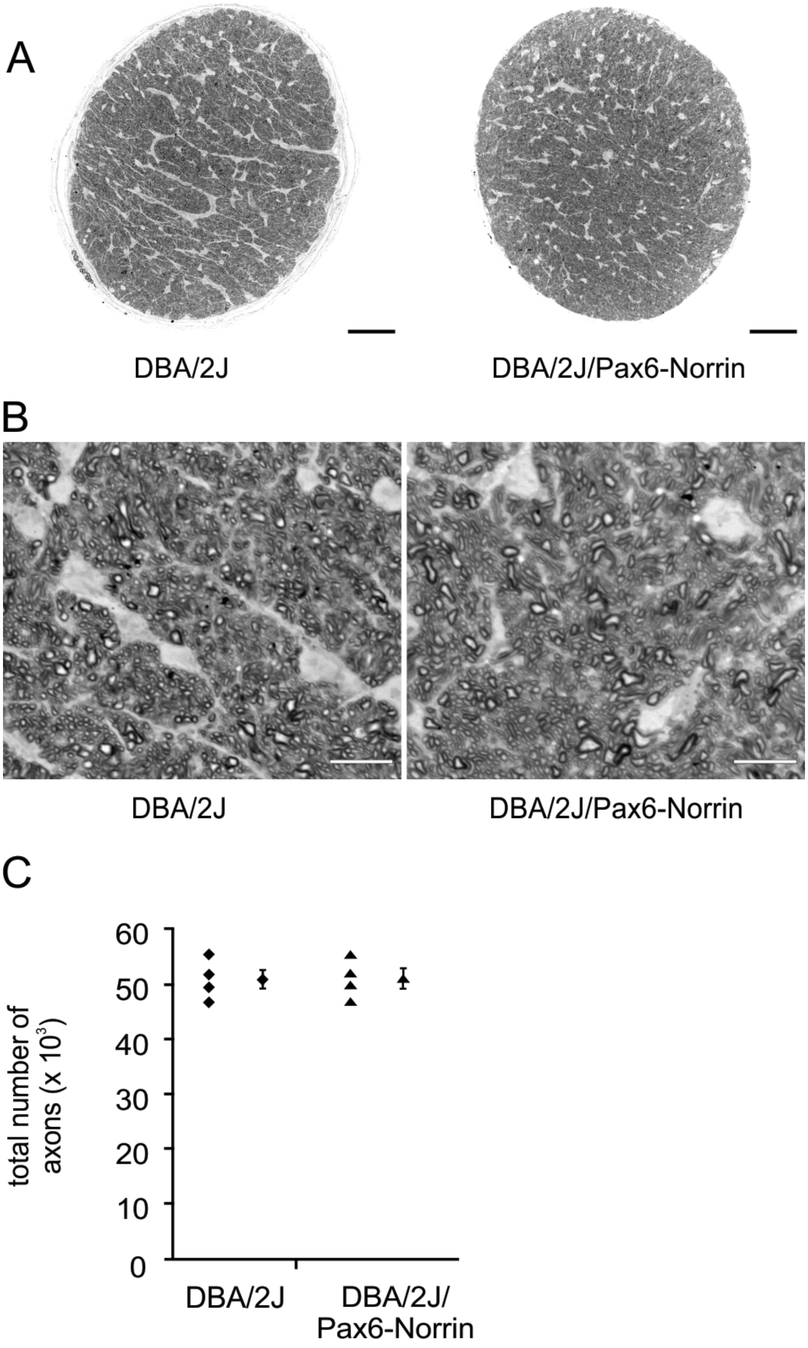

Supplemental Figure 4. Eight-week-old DBA/2J/Pax6-Norrin mice do not differ from DBA/2J littermates in optic nerve phenotype and axon number. A, B. Light microscopy of optic nerve cross sections (A) and higher magnifications (B) from transgenic eight-week-old DBA/2J/Pax6-Norrin and DBA/2J littermates (A, B). Paraphenylenediamine stain. Scale bars: A. 50 µm; B. 10 µm. **C.** For quantification, the number of axons in optic nerves from Pax6-Norrin and wild-type littermates was quantified and plotted as total number per optic nerve (mean ± SEM; n = 4).

Supplemental Figure 5

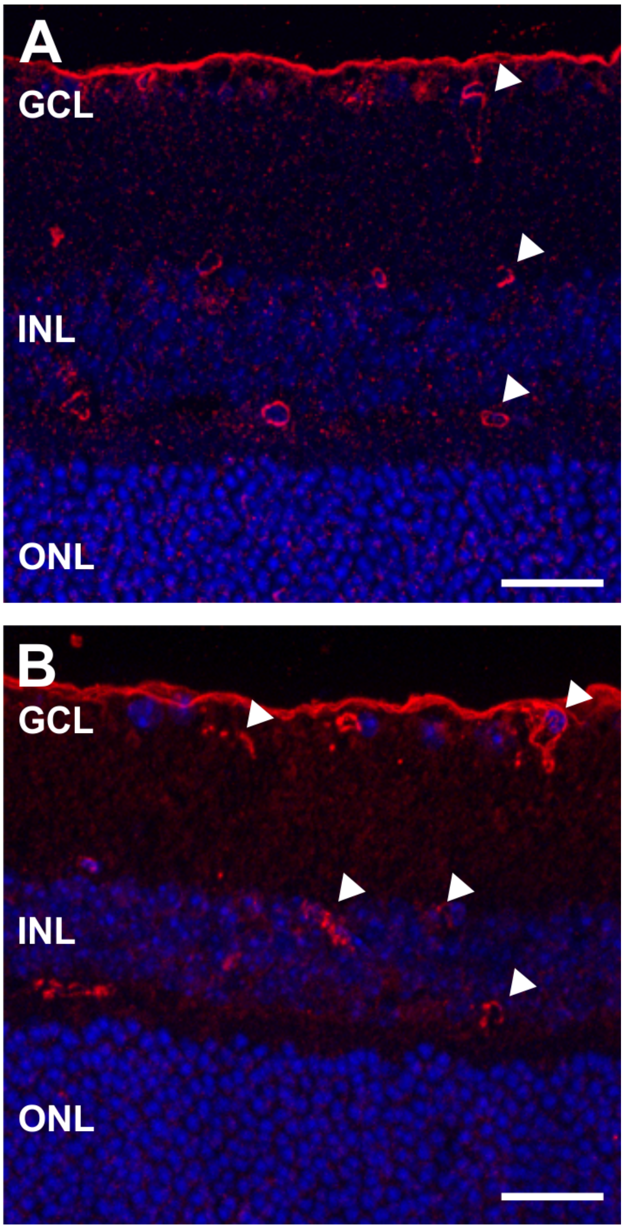

Supplemental Figure 5. Ten-month-old DBA/2J/Pax6-Norrin mice do not differ from DBA/2J littermates in phenotype of retinal vasculature.

A, B. Immunohistochemistry for collagen type IV (red) to detect the basal lamina of microvascular endothelial cells in retinae from DBA/2J (A) and DBA/2J/Pax6-Norrin mice (B) at the age of 10 months. The three vascular plexuses (arrow heads) were detected in the RGC layer, and the inner and outer edge of the inner nuclear layer of both mouse strains. Nuclei are labeled with DAPI (blue). GCL. ganglion cell layer; INL. inner nuclear layer; ONL. outer nuclear layer. Scale bars: 20 μm.

Supplemental Figure 6

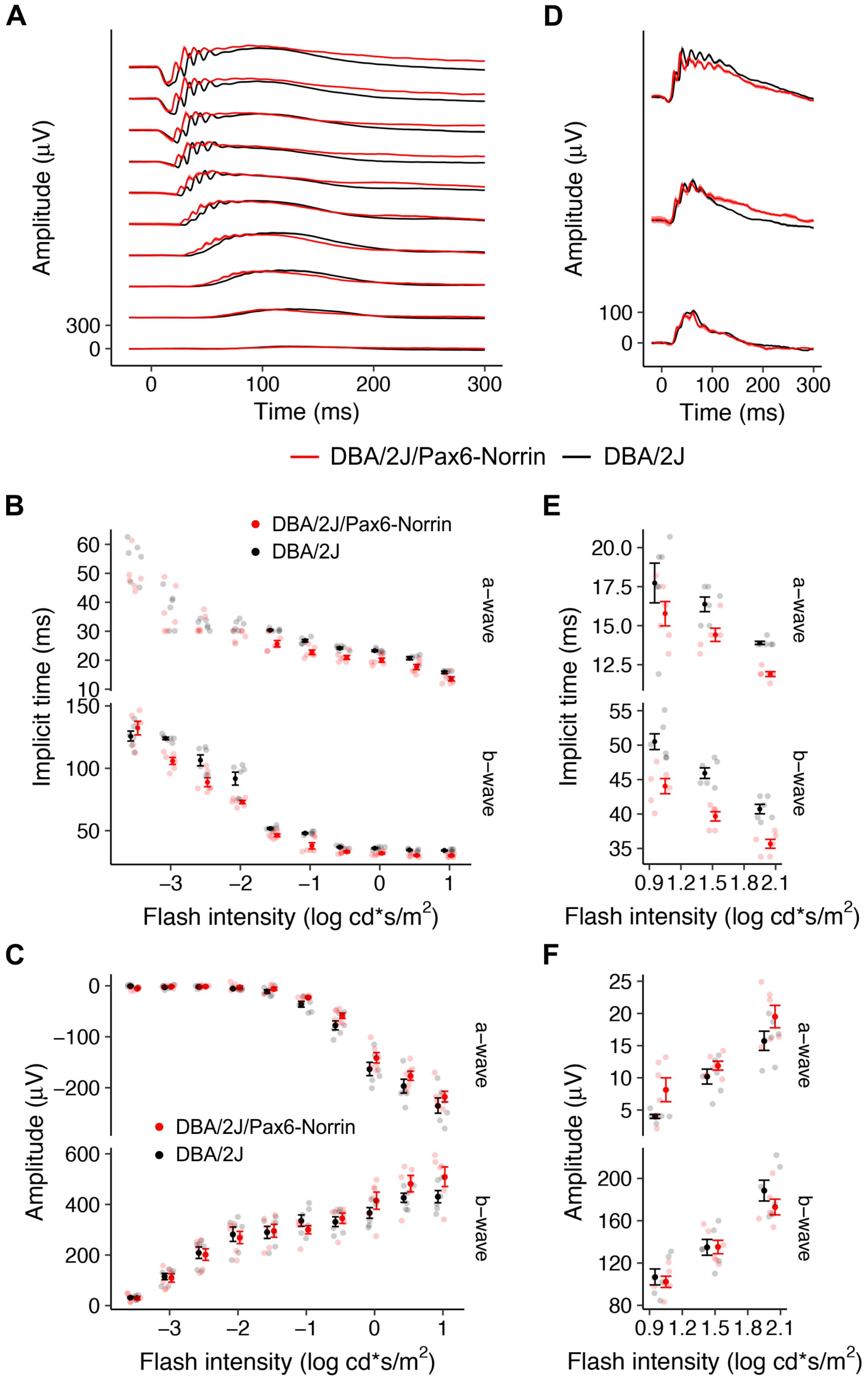

Supplemental Figure 6. ERG recordings of six-month-old DBA/2J/Pax6-Norrin and DBA/2J littermates. Single flash ERG recordings from dark-adapted (scotopic, panel A-C) and light-adapted (photopic, panel D-F) six-month-old DBA/2J/Pax6-Norrin and DBA/2J littermates (n = 3 animals). Panels A and D show average waveforms with standard error bands, while panels B, E and C, F show scotopic and photopic single flash ERG implicit times (B, E) and a- and b-wave amplitudes respectively (C, F; n = 3 animals).

Supplemental Experimental Procedures

Generation of Pax6-Norrin mice

Two fragments of the *Pax6* α-enhancer fragment were PCR amplified from murine genomic DNA using the primer pairs 5′-CTGGGAATTACCCTGGCT-3′ and 5′-GCGCGCACGCGTGTGGAATA-3′ (fragment 1; 1258 bp) and 5’-GGTAAAATCATAGACGCGCTCCTTC-3’ and 5’-TGGGCAGCCCAGCCTCAAA-3’ (fragment 2; 749 bp), and cloned into the pDrive vector according to the manufacturer's instructions (Qiagen). The endogenous *Bss*HII restriction site of both fragments was used to insert fragment 1 into the pDrive fragment 2 vector after *Kpn*I-*Bss*HII digest to obtain the *Pax6* α-enhancer fragment (1642bp). The P0 minimal promoter fragment was amplified using the primer pairs 5’-GAACCTAAGGACAGGCTACG-3’ and 5’-CATGAATTCGGCGCGAGGCTTG-3´ (990bp) and cloned into the Topo blunt vector (Invitrogen). To introduce restriction sides for *Sac*II at the 5’-end, an additional PCR with the primer pair 5’-CCGCGGGAACCTAAGGACAGGCTACG-3’ and 5’-CATGAATTCGGCGCGAGGCTTG-3’ (containing an endogenous *Eco*RI restriction site) was performed and the obtained *Sac*II-P0 minimal promoter-*Eco*RI fragment was cloned into a Topo blunt vector. Following this, an additional PCR of the *Pax6* α-enhancer promoter fragment was performed using the primer pairs 5′-GAATTCGCCCTGCGGCCGCTGGGAATTACCCTGGCT-3′ and 5′-CCGCGGTGGGCAGCCCAGCCTCAAA-3′ to introduce at the 5′-end an *Eco*RI-*Not*I and at the 3′-end a *Sac*II restriction site, and subcloned into the pDrive vector. After restriction digest with *Kpn*I and *Sac*II the *Eco*RI-*Not*I-*Pax6* α-enhancer-*Sac*II promoter fragment was cloned into the *Sac*II-P0 minimal promoter-*Eco*RI Topo blunt plasmid to obtain the Pax6 α-enhancer-P0 promoter fragment, hereinafter referred as

Pax6 promoter. All PCR products were sequenced before further cloning. The βB1-Crystallin promoter fragment of the βB1-Norrin plasmid [10] was replaced with the *Eco*RI-*Not*I-*Pax6*-*Eco*RI promoter fragment by *Eco*RI digest to obtain the plasmid Pax6-Norrin. Before microinjection, the construct was finally sequenced and released from the plasmid Pax6-Norrin by digest with *Not*I. For generation of transgenic mice, ES cells derived from FVB/N blastocysts were coelectroporated with the Pax6-Norrin expression construct and a circular neomycin resistance plasmid (PGK-Neo-bpA). G418-resistant individual colonies were isolated and screened for full-length integration of the Pax6-Norrin expression construct. Positive clones were expanded and used for the generation of chimeric mice via microinjection of recombinant ES cells into blastocysts of C57BL/6N host embryos. Chimeric offspring were mated with isogenic FVB/N females for germline transmission of the Pax6-Norrin construct and establishment of three independent mouse lines. Since transgenic Pax6-Norrin mice have been generated in an FVB/N background, carrying the recessive retina degeneration-1 (rd1) mutation, transgenic mice were bred with wild-type CD-1 mice (Charles River) and only F1 animals with a phenotypically normal retina were used throughout the phenotype analysis of FVB/N/CD-1 transgenic mice.

Northern blot analysis

For Northern blot analysis PCR was used to amplify a cDNA fragment of murine Norrin from plasmid Pax6-Norrin by using the primer pairs 5′-AGCTCAAAGATGGTGCTCCT-3′ and 5′-TAGAGCCAACAGGGGAAATG-3′ (product length, 495 bp). PCR products were gel-purified by using the Qiagen Gel purification kit (Qiagen) and cloned into pCR Topo TA vector (Invitrogen). After linearization of the vector with *Hind*III, an antisense RNA probe for Norrin was generated and

labeled with DIG-11-UTP using T7-polymerase (Roche). For Northern blot analysis, 10 µg total RNA was separated on a 1% agarose gel containing 6 % formaldehyde and blotted onto a positively charged nylon membrane (Roche). After transfer, the blot was cross-linked using a UV Stratalinker 1800 (Stratagene). Prehybridization was performed for 1 h at 60°C using the Dig EasyHyb-buffer (Roche). After overnight hybridization at 60°C, membranes were washed for 5 min with 2x SSC and 0.1% SDS at room temperature and 15 min with 0.2x SSC and 0.1% SDS at 70°C. For detection of hybridization signals, membranes were blocked for 30 min at room temperature in 1% blocking reagent, 0.1 M maleic acid, and 0.15 M NaCl (pH 7.5) and incubated 30 min in anti-digoxigenin-alkaline phosphatase (1:10,000; Roche). After washing membranes two times for 15 min in 0.1 m maleic acid, 0.15 m NaCl (pH 7.5) and 0.3% Tween 20, chemiluminescence detection was performed (CDP-Star; Roche). The membranes were visualized on a BAS 3000 Imager work station (Fujifilm). To monitor the integrity of RNA, the relative amounts of RNA loaded on the gel and the efficiency of transfer, membranes were stained with methylene blue. The intensity of the hybridization signal was determined by the Aida Image Analyzer v.4.06 software (Raytest).

Histological grading of the anterior eye segment

For semiquantitative analysis of the pathological changes, we used a grading system published previously that identifies no or mild, moderate and severe changes [21]. Changes in chamber angle structure are no or mild when anterior synechiae cover only part of the trabecular meshwork, moderate when they completely cover trabecular meshwork and the very periphery of the cornea, and severe when synechiae extend more centrally onto the cornea (Fig. 6A). Changes in ciliary body

structure are regarded as mild when the processes are shortened but epithelium and vascular structures appear normal, moderate when processes are atrophic with less distinct vascular and epithelial layers, and severe when process are not recognizable but atrophied to a flat epithelium (Fig. 6C). Iris changes are no or mild when the stroma is of normal thickness with fewer than normal iris pigment epithelium cells present, moderate when the stroma is thinner than normal and few or no pigment epithelium cells are present, and severe when the stroma is very thin and no pigment epithelium present (Fig. 6E).

Electrophysiology

Mice were dark adapted for at least 12 hours. Mice were anesthetized by subcutaneous injection of ketamine (65 mg/kg) and xylazine (13 mg/kg), and their pupils were dilated with tropicamide eyedrops (Mydriaticum Stulln). Silver needle electrodes served as reference (fore-head) and ground (tail) and gold wire ring electrodes as active electrodes. Corneregel (Bausch & Lomb) was applied to keep the eye hydrated and to maintain good electrical contact. ERGs were recorded using a Ganzfeld bowl (Ganzfeld QC450 SCX, Roland Consult) from both eyes simultaneously, band-pass filtered (1 to 300 Hz) and averaged. Single flash scotopic (dark adapted) responses to a series of ten LED-flash intensities ranging from -3.5 to 1 log cds/m^2 with an inter stimulus interval of 2 up to 20 s for the highest intensity were recorded. After 10 minutes of adaptation to a white background illumination (20 cd/m^2) single flash photopic (light adapted) responses to three Xenon-flash intensities (1, 1.5 and 2 log cds/m^2) were recorded. All analyses and plotting was carried out with R 3.3.2 and ggplot 2.2.1.
